# Assessment of the activities physiological cost of the defense forces officers in Ukraine using miniature ECG device

**DOI:** 10.3389/fcvm.2023.1239128

**Published:** 2023-10-06

**Authors:** Mykhailo Bocharov, Vasyl Stasiuk, Vasyl Osyodlo, Tetyana Ryzhenko, Vlad Malanin, Dmytro Chumachenko, Illya Chaikovsky

**Affiliations:** ^1^ Department of Moral and Psychological Support of the Activity of the Troops (Forces), National Defense University of Ukraine Named After Ivan Cherniakhovskyi, Kyiv, Ukraine; ^2^ Humanitarian Institute, National Defense University of Ukraine Named After Ivan Cherniakhovskyi, Kyiv, Ukraine; ^3^ Glushkov Institute of Cybernetics of National Academy of Science, Kyiv, Ukraine; ^4^ Department of Mathematical Modelling and Artificial Intelligence, National Aerospace University “Kharkiv Aviation Institute”, Kharkiv, Ukraine

**Keywords:** physiological cost of servicemen's activity, complex functional state index, psychoemotional state index, ultra-miniature ECG devices, heart rate variability

## Abstract

**Introduction:**

In the principles of the organization of armed struggle of the defense forces of most developed countries of the world, considerable attention is paid to the evaluation of combat readiness of the military personnel. This procedure is conditioned by such interconnected goals of the armed struggle as the maximum realization of the combat potential and the minimization of personnel losses. The purpose of the work is to determine the physiological cost of the activities of the soldiers of the Defense Forces of Ukraine with the help of miniature electrocardiographic hardware and software complexes.

**Methods:**

In the research, ultra-miniature ECG devices worn on the body for a long time, so-called wearable “on-body” ECG patch devices, were used in various combat conditions. When analyzing the data, the principle of multi-faceted ECG analysis was implemented, which allows you to obtain complete and physiologically based information, which includes 4 blocks: heart rate variability (HRV), amplitude-time indicators of the ECG, heart rhythm disorders, and psycho- emotional state.

**Results:**

In this study, a complex index of the functional state formed based on estimates of generally accepted and original indicators of heart rhythm variability, the shape of the teeth and complexes of the electrocardiogram, as well as an index of the psycho-emotional state formed according to the same principles based on the analysis of heart rhythm variability according to the modified McCraty algorithm (USA) was evaluated. Examination with the help of the complex is carried out in a state of rest, sitting or lying down.

**Discussion:**

The sensitivity of the developed monitoring system is good enough to detect the changes in the functional state both in the case of short-term (for hours) intense physical or psycho-emotional stress and more chronic (for days and weeks) stress depending on the nature of the task being done. The proposed methods and means can be considered an important tool to support the commander's decision-making regarding the ability of personnel from the point of view of their functional state to perform combat tasks.

## 1. Introduction

The lessons of the operations of the Armed Forces of Ukraine in the war against Russia (1, 2) indicate the limited resource capabilities of the parties, the absence of a clear advantage in the air, at sea, and on land, and a single acceptable strategy of actions against the aggressor—holding the territory, exhausting the enemy, using political, information and technical support of friendly countries of the world.

An analysis of the hostilities in eastern Ukraine in May and June 2022 (3, 4) indicates an increase in the intensity and density of the enemy's firepower, which leads to an increase in sanitary and psychogenic losses and a gradual decrease in the professional and physical qualities of the human resources of the Ukrainian troops.

Under these conditions, the quality of military planning, based on a realistic assessment of capabilities, including the physiological capabilities of personnel, is of great importance.

The method of behavioral analysis (methodology of visual diagnostics and observation, letters for interviewing of commanders), known since the time of the Vietnam War (5, 6), is basic in this context but involves the subjective assessment of a significant number of experts with a certain level of knowledge, skills, and abilities, therefore is not practical with the beginning of intense hostilities.

Hence, practical technologies are needed based on the registration and analysis of objective biological signals.

At the same time, the insufficient technological capacity of units of the Defense Forces of Ukraine to monitor the psychological status of soldiers has been revealed. Besides, the prejudices of the majority of the leadership of the units regarding the feasibility of soldiers' physiology monitoring, first of all, mental health, is obvious, despite the well-known fact that psychogenic losses can be up to 30% of sanitary losses (7).

The purpose of this work is to carry out long-term monitoring of the functional state of the officers of the Defense Forces of Ukraine during their performance of various official duties in wartime to determine the physiological reserve and the physiological cost of combat activity with the help of the miniature electrocardiographic device and cloud-based analytical platform.

In this study, for the first time, the possibility of assessing the physical cost of the activities of senior officers of the Defense Forces of Ukraine under various scenarios of combat activity with the help of miniature wearable ECG patch and cloud-based analytical platform. Proprietary methods and algorithms for assessing the functional and psycho-emotional state of military officers based on the analysis of subtle changes in the ECG and HRV have been developed. Various cases of long-term monitoring of the functional status of senior officers demonstrate the high and low cost of activity, depending on the nature of the task being done. The sensitivity of the developed monitoring system is good enough to detect the changes in the functional state both in the case of short-term (for hours) intense physical or psycho-emotional stress and more chronic (for days and weeks) stress. Also, the relationship between the functional state index and the psycho-emotional index was investigated for the first time. It is shown that the strength of this relationship varies depending on the type of stress.

The further structure of the paper is the following: Section 2, Current Research Analysis, provides the background of the study and brief overview of approaches on using the heart rate data during warfare. Section 3, Materials and methods, provides a description of the proposed device and proprietary software. Section 4, Results and Discussion, describes results of the experimental study and discusses the perspective use of the proposed approach. The conclusion describes the outcomes of the research.

## 2. Current research analysis

The presence of risk factors, the complication of functions, expansion of the range of controlled processes, increase in the pace of activity, the monotony of work in conditions of waiting for a signal to act, the combination of different actions in one activity (combined activity), processing of large volumes of information, the lack of time to perform the necessary actions allow us to define the activities of members of the defense forces of Ukraine in the war against the Russian Federation as highly extreme.

In the principles of the organization of armed struggle of the defense forces of most countries, considerable attention is paid to this issue. Such interconnected goals of the armed struggle as the maximum realization of the combat potential and the minimization of personnel losses condition this procedure.

The analysis of the governing documents of the structures of the security and defense sector confirms the absence of uniform approaches to the evaluation of the psychological state of the personnel of the troops, which requires the study of appropriate methods in the armed forces of other countries of the world.

The results of the analysis of modern scientific literature (8–12) in the field of military psychology indicate four components of the psychological readiness of soldiers to perform combat missions and tasks: motivational, emotional-volitional, personal, and functional. At the same time, the functional component has the most significant applied value: it is the ability to calculate the resources of one's forces concerning the forces and means of the enemy, knowledge of tactical methods of combat, physical endurance (extended stay in a confined space, long-term work in conditions during intensive combat operations and in monotonous periods activities (for example, in the case of redeployment), integration of all resources for the performance of professional activities.

The US Army developed the general approach to monitoring the moral, psychological, and physiological capabilities of soldiers in the armed forces of NATO countries during the Vietnam War. It standardized it in the relevant conceptual documents (13).

The concept of the “functional state” of the human body is often used to characterize the general condition of a person related to the performance of labor and educational activities and being in a specific moral and psychological environment which is highly relevant during its change, or changes in the conditions of its implementation (14). There are many definitions of functional status. The following seems the most successful to us: the functional state of the organism is an integral characteristic of the state of health, which reflects the level of functional reserve that can be used for adaptation.

Functional status is the ability to perform official duties. Of course, the functional state of a person changes over time. As a result of physical or mental stress, it can deteriorate. That is, the functional reserve decreases. This reduction of the reserve is a physiological price of the activity.

Many techniques and ways of assessing a person's functional state, from questionnaires to hardware and software diagnostics, are known. Regarding the requirements for assessing the functional state, in the majority of the armed forces of NATO countries, the object of assessing and predicting the functional state of service members is defined as psychological resilience, psychological resilience for the operational determination of which the statutory documents provide for convenient assessment parameters, which allows automating the collection given by personnel without special training (15). Unit commanders, junior commanders, medics, chaplains, mentors-soldiers, comrades-in-arms, family members, medical service specialists, psychologists, and their assistants—trained soldiers carry continuous monitoring of maladaptive phenomena of combat stress.

The priority of monitoring the psychological health of soldiers is gradually shifted from the command to the medical specialists of the unit according to the level of deterioration of the functional state of a person. Twice a year, the US Army assesses the psychological and physical condition of military personnel using the Global Assessment Tool GAT 2.0 (16), which integrates psychometric methods in combination with the results of physiological functional tests and the results of the Army Physical Fitness Test (APFT) (17).

An Online GAT 2.0 system is used for the individual self-development program of military personnel, civilian personnel, and their family members. Each aspect (dimension) of the online assessment is based on valid psychometric techniques in four main domains: emotional, social, family, and spiritual.

The peculiarity of the Canadian approach in applying this program is the spread of its use in other professions, in which there is a factor of psychological stress, namely: search and rescue service, particular purpose police units, and medical personnel (18).

Therefore, thanks to the integration of the results of the assessment of the condition of service members with electronic personnel databases, the army command can quickly assess the results of training, exercises, and the condition of military units before and after the combat mission, which helps in making operational decisions of combat management. The technologies mentioned above, which are based on self-assessment questionnaires, can be improved by taking into account the implementation of technologies for objective monitoring of the functional state of a person in the practice of psychological and medical support of combat operations.

Significant attention is paid to the instrumental monitoring of personal combat readiness solution of this task in the armies of NATO countries, including in the framework of long-term programs. Research is primarily conducted in units of highly mobile airborne troops and special operations forces. Monitoring was carried out using the analysis of various physiological signals, including the heart's electrical signals, namely the analysis of HRV.

There are also a number of studies on the use of electronic heart signals to assess the condition of soldiers.

The study (19) investigated whether overnight HRV reflects workload and stress during military training. The researchers assessed cognitive load, perceived exertion, physical activity, nocturnal HRV, cognitive performance, and sleep for 15 days in 32 combat engineers. The results indicated that the FIELD phase, which involved sleep deprivation and restriction, was associated with increased mood disturbance, perceived exertion, physical activity, HRV, and decreased sleep quantity. However, measures of HRV returned to pre-training values quicker than subjective well-being responses. Sleep duration and physical activity were identified as the primary stressors of the exercise, and the best predictive model of HRV included both factors. The study suggests that HRV can be a useful measure for monitoring stress during military training, and recovery rates of HRV and subjective well-being may differ.

L.W. Ko, with colleagues (20), discusses the development of a mobile EEG and ECG integration system for monitoring soldiers' physiological states during simulated war game training. The system aims to measure the soldiers' attention, fatigue, stress, emotion, and heart rate through real-time signal processing implemented on a mobile platform. The study suggests that the proposed integration system is feasible for understanding the soldier's physiological states during military training and can help monitor their performance in the future.

C.E. Bedford, with colleagues (21), examines the physiological reactivity to negative affective stimuli in active duty soldiers with chronic pain conditions who are receiving long-term opioid therapy and also have posttraumatic stress disorder (PTSD). The study analyzed heart rate and skin temperature responses during a practical picture-viewing task for 30 participants. Results indicate that soldiers with PTSD exhibit more significant increases in the ratio of low-to-high frequency heart rate variability (LF/HF HRV) when viewing negative affective images compared to soldiers without PTSD. PTSD symptom severity was positively associated with LF/HF HRV reactivity and negatively associated with skin temperature reactivity. Opioid craving was also associated with LF/HF HRV and skin temperature reactivity among soldiers with PTSD. The study highlights the importance of intervening in potential risk factors for comorbid chronic pain and PTSD in military populations.

The paper (22) describes the development of Doppler radar heart sensing technology that can detect life signs, respiration, and/or heartbeat, at a distance, even for subjects lying motionless, e.g., unconscious subjects, wearing body armor, and hidden from direct view. This technology can deliver heart rate information with high accuracy, enabling the assessment of a subject's physiological and psychological state based on HRV analysis. This can help triage wounded soldiers on the battlefield, allowing medics to prioritize which soldiers to attend to first. The paper discusses the software and hardware developments and challenges in implementing this technology, including heart signal detection from all four sides of the human body, detection in the presence of body armor, and the feasibility of HRV parameter extraction.

M. Anuradha, with colleagues (23), proposes a system for secure communication and emergency alert for soldiers during warfare, using ECG as a biometric feature. The system involves a walkie-talkie with a wireless sensor network and IoT-based monitoring to detect ECG and connect with the control station based on the soldier's pulse rate. The system also has an inbuilt GPS to identify the soldier's location. The system aims to enhance security through biometric authentication, using ECG, which is unique to each individual and cannot be misused after the person is dead. The control station receives an update message from the soldier's walkie-talkie every 2 min and alerts the head of the battalion in critical situations.

The study (24) examined the relationship between pilot mental workload (PMWL), heart rate (HR), HRV, and performance during instrument approaches in a high-fidelity simulator. The results showed that HR and HRV could distinguish the level of PMWL and differentiate sub-standard performance approaches from high-performance approaches. Therefore, HR and HRV can be used as measures of PMWL and to assess pilots' ability to cope with increasing task demands in a fighter aviation environment.

The study (25) aimed to analyze the effect of experience and training on soldiers' psychophysiological response, attention, and memory in combat situations. The research involved 49 soldiers from the Spanish Army who underwent a combat simulation. The study found that combat simulation significantly increased blood lactate, blood glucose, blood oxygen saturation, rated perceived exertion, heart rate, and cognitive and somatic anxiety. Experienced and highly trained soldiers showed a significant increase in the low-frequency domain and a significant decrease in the high-frequency domain of heart rate variability. The study also found that the stimulus nature modulated soldiers' memory function, and higher psychophysiological activation correlated positively with cognitive impairment and lower memory.

The paper (26) investigated the association between pre-deployment HRV and post-deployment PTSD symptoms in Army National Guard soldiers. The study analyzed 343 soldiers enrolled in the Warriors Achieving Resilience study, measuring HRV predictor variables and PTSD symptom severity at baseline, 3- and 12-month post-deployment. The results showed lower pre-deployment HRV was associated with higher post-deployment PTSD symptoms. The study suggested that pre-deployment HRV could predict post-deployment PTSD symptoms.

D. Patton and K. Gamble (27) examine the effectiveness of using a 300-degree immersive simulator to train US Army Soldiers in a stress-inducing environment. The study used a Shoot-Don't-Shoot task with two types of performance feedback to measure arousal levels through HRV. Higher arousal levels were seen in the Shock condition compared to the Life Bar condition. Interbeat interval (IBI) was also examined in Baseline and Post-Shock/Post-Life Bar sessions. Results showed that IBI returned to near Baseline levels after both conditions, indicating a recovery from arousal induced during the scenarios. The study demonstrates the value of objectively measuring physiology to assess heightened arousal during Soldier-relevant tasks in a simulated environment, which can indicate how immersive or stressful an environment is and its potential effectiveness as a pre-deployment training environment.

The paper (28) investigates the relationship between HRV and PTSD in a large cohort of male active-duty Marines before combat deployment. The study found that lower levels of high-frequency HRV were associated with a diagnosis of PTSD when adjusting for covariates, including traumatic brain injury (TBI) and depression symptoms. Furthermore, Marines with deployment experience had lower HRV than those without experience. The study suggests that HRV may be a valuable tool to predict vulnerability and resilience to combat exposure's psychological and physiological consequences.

A. Minassian with colleagues (29) investigated the relationship between HRV before combat deployment and the risk of PTSD development after deployment, accounting for deployment-related combat exposure. The study included 1,415 male Marines (59 with PTSD) in the first phase and 745 (25 with PTSD) in the second phase. HRV was measured via finger photoplethysmography during a 5-min rest period before deployment. Results suggest that an increased low-frequency (LF) to high-frequency (HF) ratio of HRV was associated with a higher risk of PTSD diagnosis after deployment, independent of other key risk factors, such as deployment-related combat exposure. These findings provide modest evidence that autonomic nervous system functioning contributes to PTSD vulnerability, which may open new avenues for PTSD prevention and treatment.

G.F. Lewis, with colleagues (30), investigated the relationship between HRV, PTSD, and depression symptoms in military personnel. The study randomly assigned a large sample of military personnel to either an experimental group receiving HRV biofeedback-assisted relaxation training or a control condition. Results showed that experimental subjects exhibited greater HRV and less arousal during a post-training combat simulation designed to heighten arousal. Autonomic reactivity was also related to PTSD and self-reported use of mental health services. The study concludes that HRV biofeedback-assisted relaxation training could effectively reduce psychological harm following trauma exposure by increasing the capacity for parasympathetically modulated reactions to stress and providing a coping tool for use following a stressful situation.

The paper (31) discusses using HRV to monitor stress in the military and other first responders under workplace conditions. The study finds that these populations show lower HRV during stress than at baseline and more significant post-stress rebound, controlling for potential confounders. The study highlights the need for controlling potential covariates to understand the relationship between HRV and stress response and provides a basis for hypothesis-driven research. The findings emphasize the link between stress and relaxation breathing in respiratory sinus arrhythmia and low-frequency HRV.

The study (32) investigates using overnight HRV as a repeated measure of allostatic load in defense personnel during basic military training (BMT). 48 recruits were studied, and HRV, physical performance, subjective well-being, and physical activity were measured weekly during BMT. Results showed that HRV was elevated in weeks 7 and 10. The best predictive model of HRV included perceived exertion, subjective fatigue, the number of awakenings during sleep, and changes in VO2 peak. HRV was predicted by subjective recruit responses to BMT workloads rather than objective measures of physical activity. Monitoring HRV and HRV concerning interbeat interval length may provide a better tool for determining allostatic load than HRV alone.

The paper (33) examines the importance of measuring pilot mental workload (PMWL) during an instrument flight rules (IFR) proficiency test using heart rate (HR) and heart rate variation (HRV). The study involved fighter pilots taking an IFR proficiency test in a F/A-18 simulator. The results showed that HR and HRV could differentiate varying task demands and can be used to measure PMWL during the test. The study concludes that measuring PMWL during a proficiency test is essential and should be done with task performance measurement.

Thus, the analysis showed the absence of studies to which this article is directed. In addition, none of the studies was conducted in combat conditions but described experimental studies, simulators, and military training.

## 3. Materials and methods

Five senior officers (male, mean age 39 ± 4 years) were examined with the help of wearable “on-body” ECG patch devices (Solvaig LTD, Kyiv) under various combat situations.

**Figure 1:**
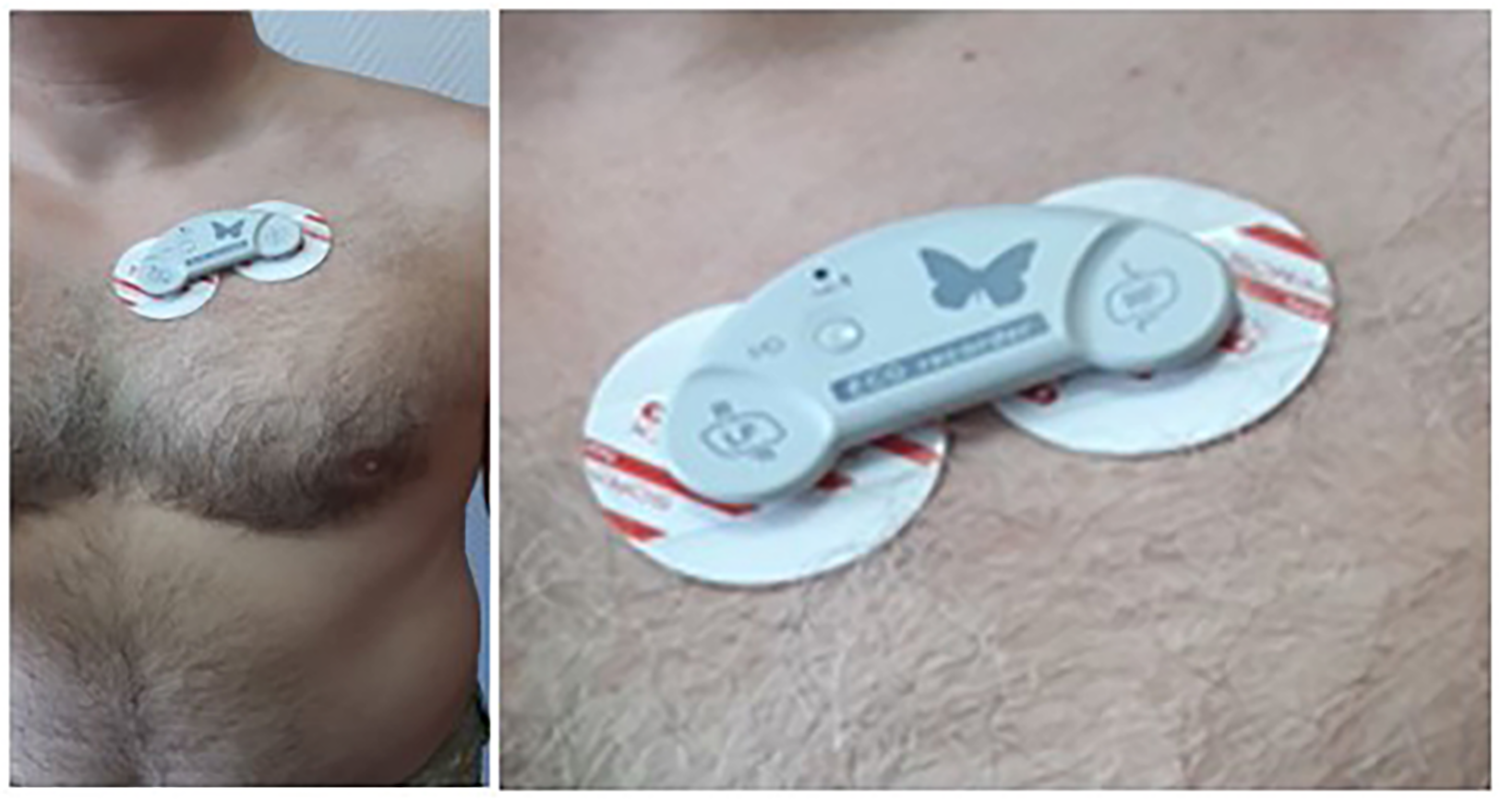
ECG “Butterfly” patch recorder—mounting under the left collarbone.

**Figure 2:**
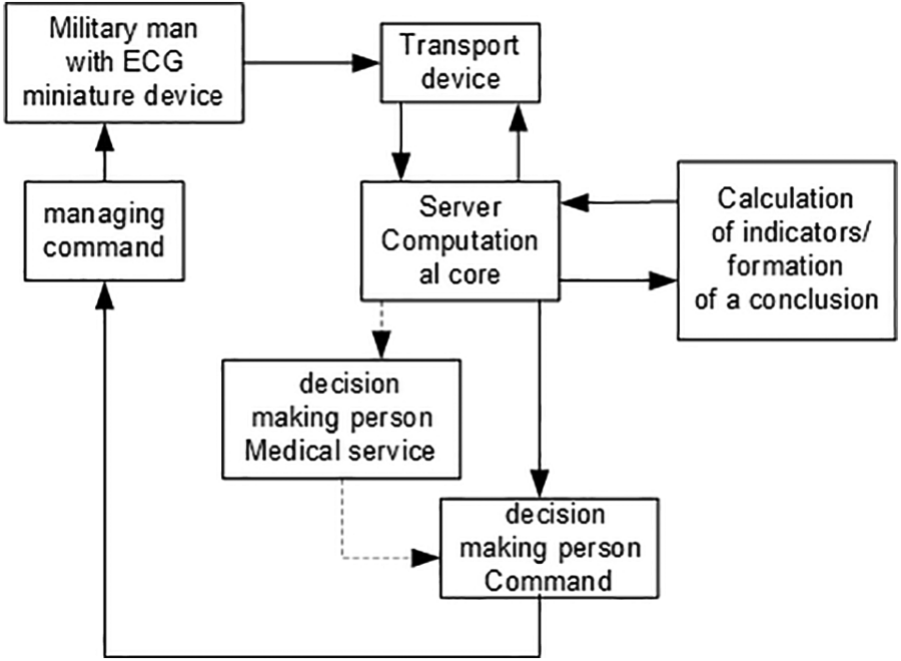
Technological idea and architecture of the information system for defense forces officers monitoring.

These ECG recorders are self-powered portable devices designed for 1-lead or 6-lead ECG recording. The recorders can work in two modes: “on-line” electrocardiograph—a set-top box (Figure 1) that registers, converts, and transmits the ECG signal to an externally controlled device (ECD) in real-time; “off-line” ECG recorders—recording is performed in the internal memory with further transfer to the ECD for visualization, storage, printing and sending to cloud services for further storage and processing. The information system's technological idea and architecture are presented in Figure 2.

These ultra-miniature and ultra-light wearable devices, which are attached to a convenient place on the body (usually under the left collarbone) with the help of sticky electrodes, do not interfere with a person's normal activities and allow monitoring of the electrocardiographic signal for many days.

Another essential technology component is protected data storage based on “cloud” technologies with appropriate cyber security certificates. The computing “core” located in such a “cloud” (that is, a set of appropriate algorithms) performs the analysis of the electrocardiogram. The Ukrainian-Finnish company Cardiolyse Oy developed this cloud technology.

**Figure 3:**
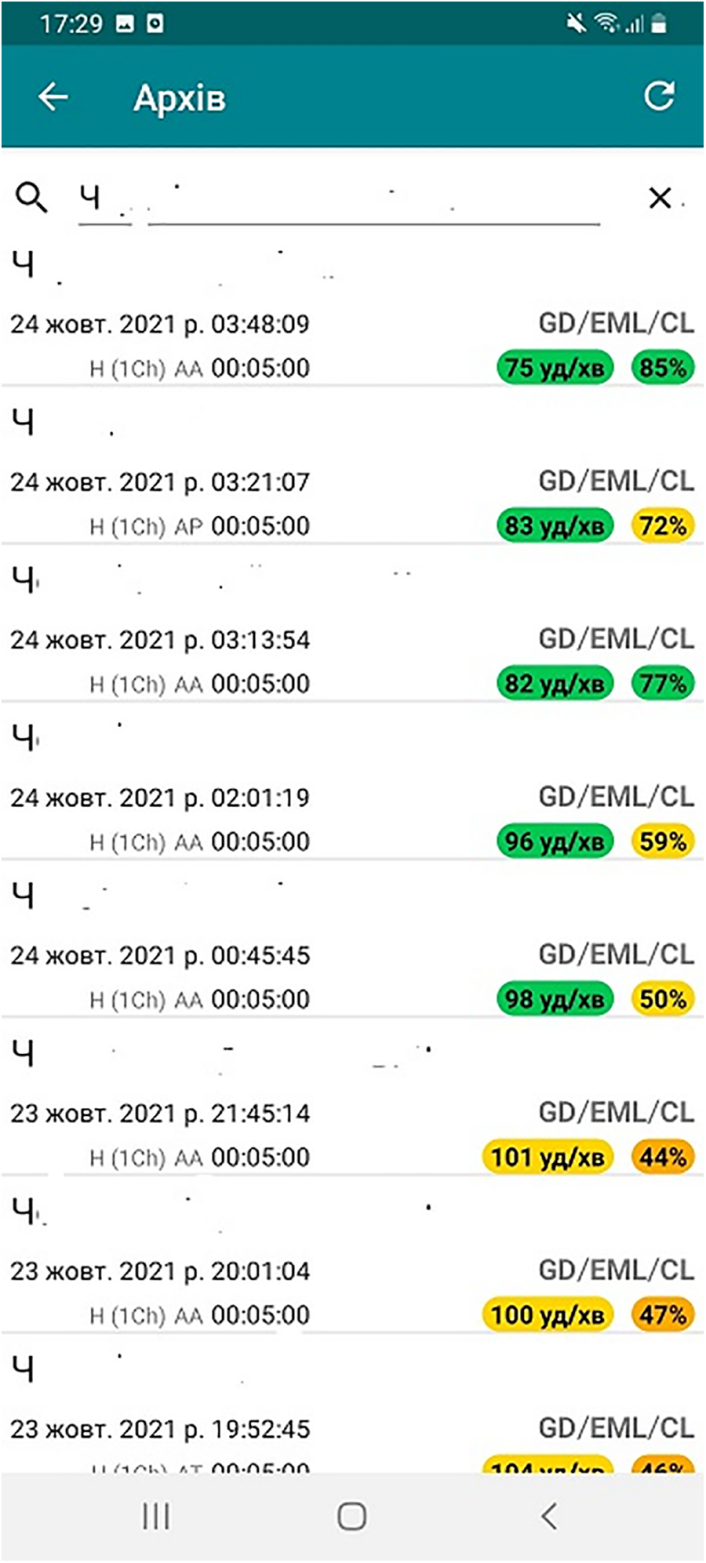
An example of infographics on the screen of a smartphone: indicators of the functional state and the heart rate (dynamics over one night).

In these software products, the principle of multi-level ECG analysis is implemented, which allows you to obtain complete and physiologically based information, which includes 4 blocks: heart rate variability (HRV), amplitude-time indicators of the ECG, heart rhythm disorders, and psycho-emotional state (34). The appropriate infographic display according to the rules of cognitive logic significantly improves the perception of the evaluation results (Figure 3).

The architecture of this proprietary software is the following.

A multilevel analysis of electrocardiogram and heart rate variability was developed according to the hierarchical principle. This analysis consists of four levels, which are listed in ascending order.

(1) The first (lower) level consists of a set of individual indicators that describe various aspects of heart rhythm variability (including psycho-emotional state), amplitude-time parameters, as well as the shape of ECG waves, the presence of major heart rhythm, and conduction disorders.
(2) The second level consists of related indicators with a close physiological meaning. Some of these groups reflect, to a greater extent, operational, that is, the immediate functioning of the cardiovascular system. These groups of indicators characterize the immediate adaptive response to external stimuli. Other groups of indicators reflect the level of the functional reserve to a greater extent.
(3) The third level is represented by three integral blocks, each of which reflects different aspects of the functioning of the cardiovascular system based on hardware analysis of the ECG. These are blocks for assessing regulation, the condition of the myocardium, and the severity of heart rhythm disorders.
(4) The fourth, highest level is a general integral indicator of the functional state of the cardiovascular system. At the same time, at higher levels of analysis, the information obtained at the previous level is summarized and aggregated. This is expressed by averaging the point values of all parameters of the indicators of the previous level. That is, the indicators of the first level are averaged at the second level, the second—at the third, and the third—at the fourth. Color coding of gradations of the functional state is used to visually display the results following the principles of the so-called slightly modified “traffic light logic”. In other words, the range of normal values is colored in green, the range of minor changes—in yellow, and significant changes—in orange. The fourth range, the range of pronounced changes, is colored red.

In the proposed concept, the range of normal values refers to the quantitative limits of the body's functioning, which are a standard, a benchmark. Median values of the normal range of indicators are estimated by the maximum number of points (90–100) and values closer to the limit of this range—by a slightly smaller number of points (76–89). Values outside the normal range are scored depending on the “distance” to the median value. The optimal number of ranges is exactly four: the norm, minor changes, essential changes, and gross changes. In the adaptation theory, these four ranges are called, respectively, the satisfactory state, the tension of adaptation, the unsatisfactory state, breakdown of adaptation.

The “functional state” of the human body is often used to characterize a person's general state, related to the performance of labor and educational activities and being in a specific moral and psychological environment. It is logically interpreted as the ability to perform official duties or the integral characteristic of health, reflecting the functional reserve level that can be used for adaptation. Of course, the functional state of a person changes over time. As a result of moral, physical, or mental stress, it can deteriorate. That is, the functional reserve will decrease. This reduction of the reserve is a physiological cost of an activity. The more significant the reduction of the functional reserve during activity, the higher the physiological cost is paid.

The complex index of the functional state is formed based on estimates of generally accepted and earliest indicators of the variability of the heart rhythm, the shape of the waves, and the electrocardiogram complexes. The psycho-emotional state index is formed according to the same principles and is determined based on the analysis of heart rhythm variability according to the modified McCraty algorithm (USA) based on a model of the so-called neuro-visceral integration (The Model of Neurovisceral Integration) was developed (35).

A thorough experimental operation of the developed proprietary complex in clinical and non-clinical conditions, including among military personnel in the combat zone revealed its capabilities in pre-hospital diagnostics, that is, providing an operational assessment of the physiological potential (reserve) of military personnel (including a cadet or a student of a military educational institution), determination of its combat capability (operational combat readiness as well as under conditions that require emergency assistance (34).

Determining the physiological cost of an activity based on objective indicators is based on the principles outlined by Chaikovsky (36). Under the physiological cost, we understand the reduction of the functional reserve during the activity. The more significant the reduction of the functional reserve during activity, the higher the physiological cost is paid.

## 4. Results and discussion

We present the results of monitoring five senior officers of various ranks (major, colonel-lieutenant, colonel) involved in the daily round-the-clock protection and defense of objects in firing positions and the performance of secondary organizational and economic organizational tasks related to the provision of combat operations.

Monitoring began two weeks after the large-scale invasion. The integral indicator of the functional state and the psycho-emotional state index were evaluated.

**Figure 4:**
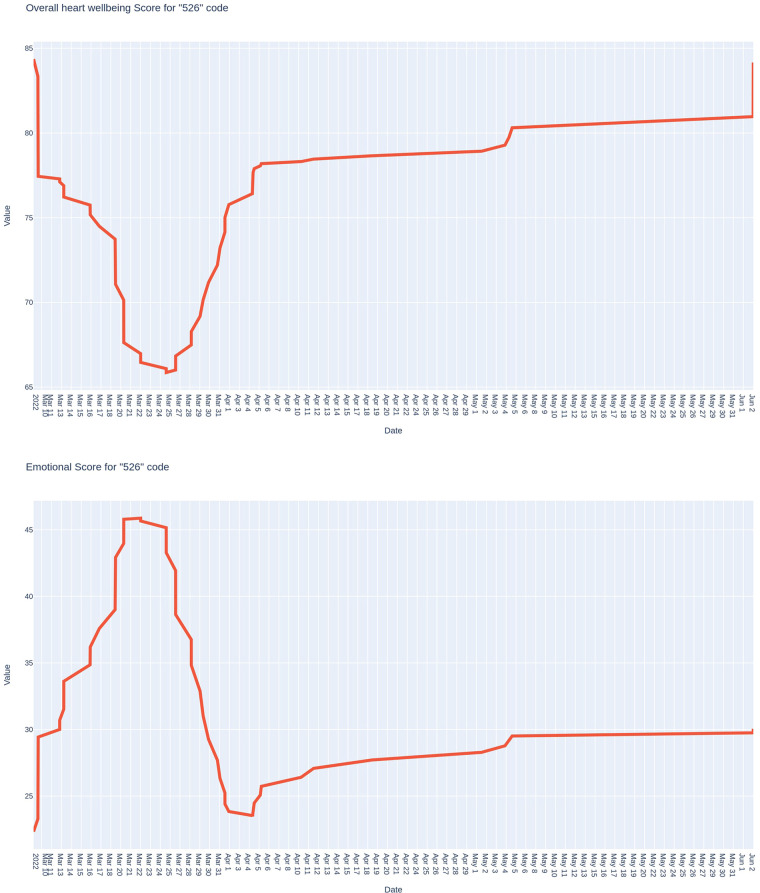
The trend of the integral indicator of the functional state and the index of the psy-cho-emotional state of the middle-ranking officer-manager.

In the below case, we give an example of the dynamics of these indicators for an officer who performs the functions of a middle-level manager. Figure 4 shows the dynamics of the integral indicator of the functional state and the psycho-emotional index.

As it is presented on the graph of the integral indicator of the functional state, at the beginning of the observation period, there are relatively high indicators of the functional state (around 85 points), which gradually decrease, creating a kind of “pit” a week after the start of monitoring (around 65 points). This level was maintained for about 1 week and then gradually recovered to approximately 80 points, at which it remained for quite a long time. The beginning of the above-mentioned “pit” of the functional state coincides with the end of the grueling regime of performing an unbalanced schedule of daily alternations. After optimizing the daily shift schedule, the service mode became more gentle. Interestingly, the emotional state index values were somewhat inverse to the integral index.

Generally speaking, during the entire observation period, they were lower than the same officer in the pre-war period. At the beginning of the observation period, the values of this index were the lowest (around 25 points). Within a week, it rose to values of around 45 points, returned to the level of 25 points for a short time, and gradually increased during the following month.

Therefore, according to the assessment results and the index of the psycho-emotional state of servicemen on the example of a middle-ranking officer, the sufficient sensitivity of the software and hardware complex to monitor psycho-emotional loads under the new conditions of service and combat tasks in wartime was determined. Periods (between March 12 and 22) were determined when the physiological cost of this officer's activity was too high, creating the prerequisites for the adaptation disruption.

**Figure 5:**
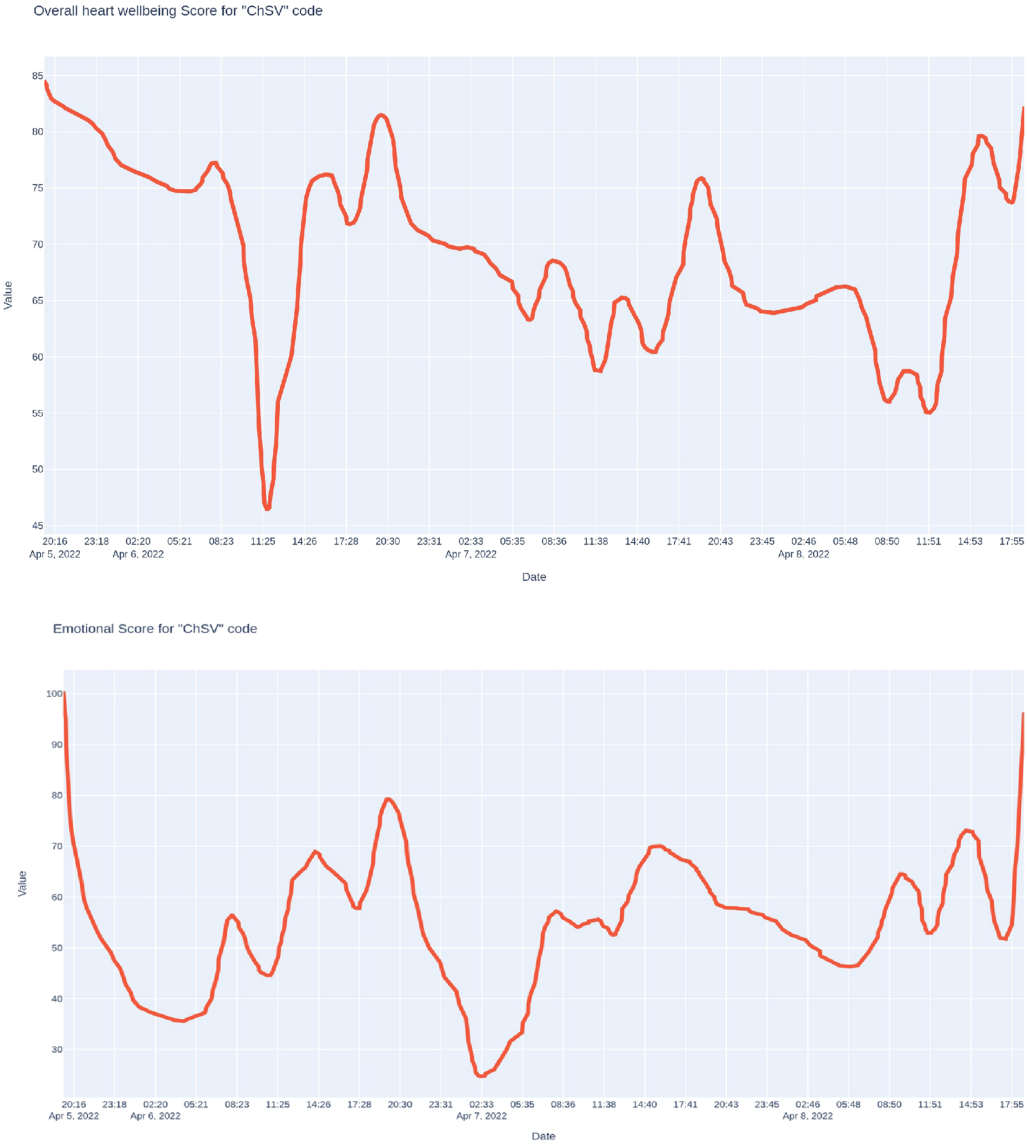
Trends of the integral indicator of the functional state and the index of the psy-cho-emotional state of a soldier of the defense forces of Ukraine before and during his visit to the personal housing destroyed by the occupiers in the city of irpin immediately after his release.

The following example reflects the 3-day monitoring of an officer of the Defense Forces of Ukraine before and during his visit to a personal residence destroyed by the occupiers in Irpin immediately after its release (Figure 5).

The first monitoring day was dedicated to preparing for the trip to Irpin. We observe a short-term sharp decrease in the integral indicator of the functional state around noon during intensive physical training. In the second half of this day, the integral indicator returned to the initial level—around 85 points. In the evening hours, this indicator decreased somewhat. The psycho-emotional state indicator was unstable—it was pretty low at the beginning of the night, increased substantially in the morning and during the day, especially in the afternoon, and decreased late in the evening. During intense physical exertion, the demonstrable psycho-emotional state also decreased somewhat for a short time, but only for a short time and to a small extent. On the second day of monitoring, we can see a smooth decrease of the integral indicator of the functional state at the beginning of the night, its improvement in the morning, again a smooth decrease in the first half of the day, and a substantial increase in the afternoon, which was replaced by a decrease in the late evening. The psycho-emotional state indicator was characterized by a sharp decrease during the night, an increase in the early morning, and a plateau with a tendency to decrease from seven to eleven in the morning, precisely when this officer was involved in assessing the severe consequences of the occupation in the city of Irpin, then a substantial increase until the 17th hours, and again a smooth but substantial decrease until the end of the day. On the third day, monitoring was carried out until 5 p.m. The dynamics of both indicators resembled the previous day. It must be said that the diary of events that this soldier kept during monitoring confirms that all three nights were, for various reasons, mainly sleepless and related to emotional experiences.

Thus, the applied monitoring system is sensitive to changes in the functional state in the case of short-term (for hours) intense psycho-emotional stress.

**Figure 6:**
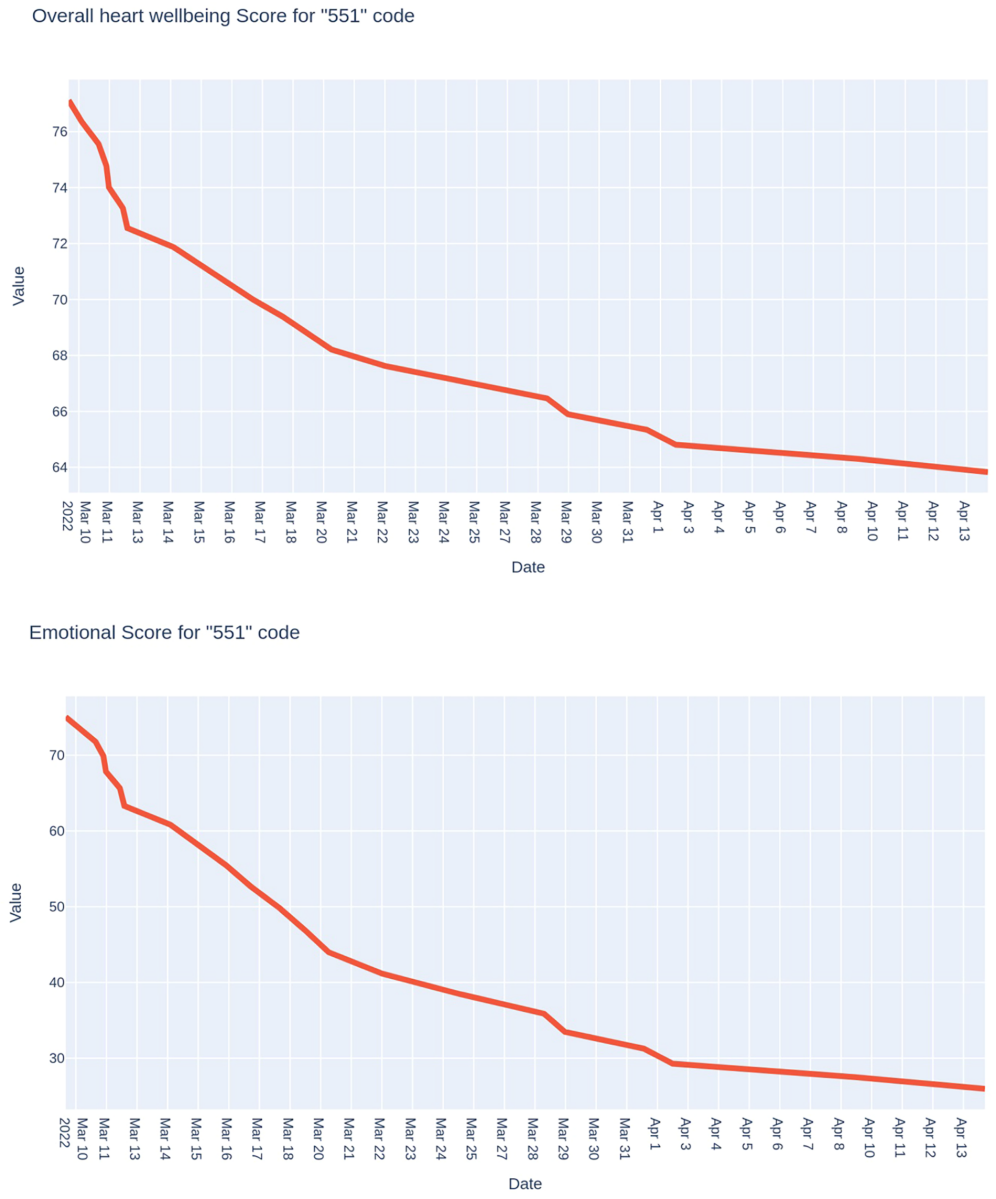
Trends of the integral indicator of the functional state and the psycho-emotional state index of the leader, the commander of the combat unit.

The third example is the monitoring results of the chief officer, the combat unit commander (Figure 6). It was carried out for one month, from mid-March to mid-April. We see a smooth decrease in both indicators: from approximately 75 points to 65 points in the case of the integral indicator of the functional state and, even more significantly, to 40 points in the case of the psycho-emotional state indicator.

As you can see, at the beginning of the studied period, the physiological price of the commander's activity was relatively low. At the end of the month of research, it increased and became average.

**Figure 7:**
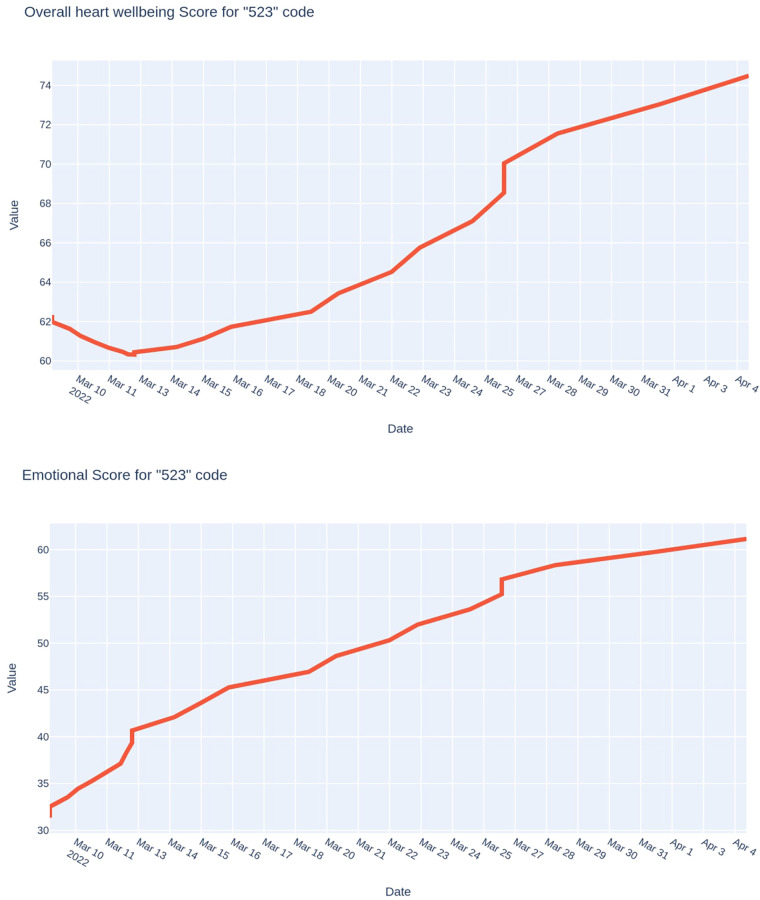
Trends of the integral indicator of the functional state and the psycho-emotional state index of a officer of the defense forces of Ukraine, whose family was in the occupied settlement for a certain time.

The following case (Figure 7) demonstrates the results of monitoring also during one month of an officer of the Defense Forces of Ukraine, who was under the influence of severe stress as his family was in a temporarily occupied settlement in the Kyiv region. Compared to the previous case, we can see the opposite trend: a smooth increase in the values of both indices that were studied. The dynamics of the psycho-emotional state index are particularly characteristic: it was extremely low (around 25 points) at the beginning of the research period. It increased to 60 points after the liberation of the occupied settlement and reunification with the family.

In this case, the physiological cost of activity during the observation period decreased—from average to low.

**Figure 8:**
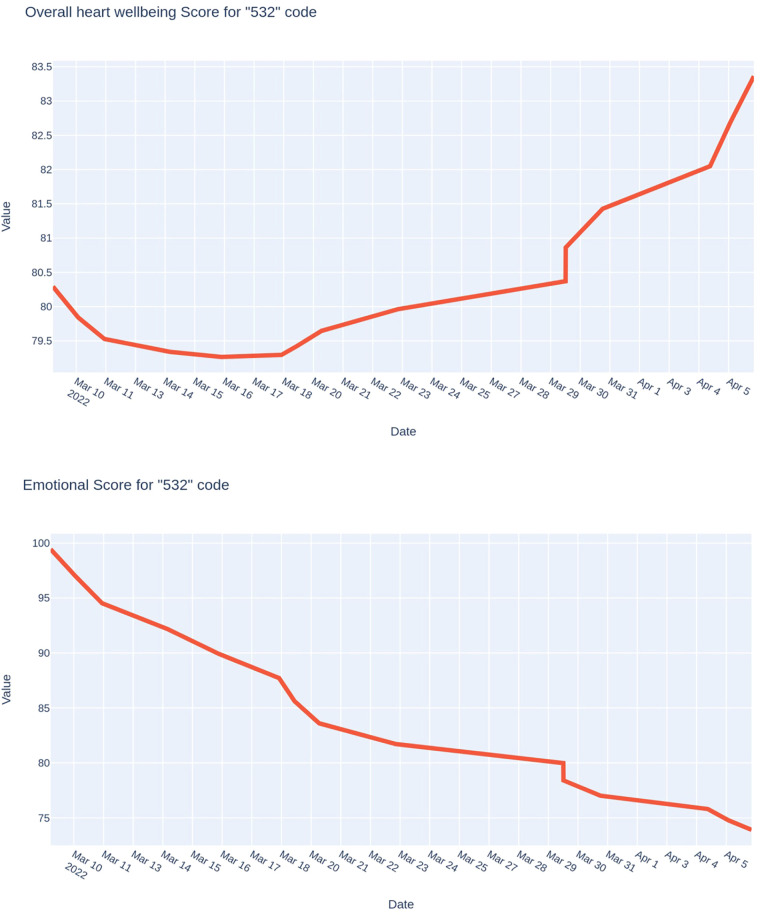
Trends of the integral indicator of the functional state and the psycho-emotional state index of a soldier who performed only routine functions.

Finally, the last example (Figure 8) shows the results of monitoring a soldier who performed only routine duties during the entire monitoring period. As you can see, both indices were much more stable than in previous cases. The integral index changed by only 2–3 points, and the psycho-emotional state index slightly worsened but remained relatively high (about 75 points).

In this case, the physiological price of activity during the observation period changed insignificantly.

There is a question about the relationship between the indexes we studied: the independence of the information contained in them. We calculated the Pearson correlation coefficient between these two parameters to solve this issue. It was found that in the above examples 3 and 5, which were not associated with rapid and sharp changes in the psycho-emotional state, the correlation between the integral indicator and the psycho-emotional state indicator is quite significant, around 0.7–0.75. On the contrary, in cases 2 and 4, where such changes occurred, there was practically no correlation. The Pearson coefficient was only 0.2. In intense, sharp psycho-emotional stress cases, it is necessary to analyze both parameters separately. Case 1 is particularly interesting, where we observe a strong but negative correlation (−0.8) between the studied parameters. This phenomenon deserves further study.

The technology described above can monitor military officers' moral, psychological, and physiological qualities through the joint efforts of specialists in medical, social, and humanitarian support. Further research will be aimed at confirming and further developing the obtained results by examining a larger group of military personnel.

In the course of future research, an algorithm will be developed for the use of dynamic assessment of the functional state and the physiological cost of the activity in the management decisions of the command, including a clear division of responsibilities between the command, psychological and medical services.

## 5. Conclusions

The practical implications of this study are profound. It underscores the feasibility of ascertaining the physiological cost of activities undertaken by senior officers of the Defense Forces of Ukraine under diverse combat scenarios. This is achieved using a miniature wearable ECG patch and a cloud-based analytical platform. By monitoring the functional and psycho-emotional state of military personnel, we can provide an objective measure to determine the individual combat readiness of each officer. This not only aids in task assignment but also helps preempt a potential breach of their physiological and functional thresholds, thereby setting the stage for maladaptation.

From a scientific perspective, the sensitivity of the monitoring system developed in this research is commendably adept at detecting shifts in the functional state. This holds for short-term intense stressors, be it physical or psycho-emotional, and more chronic stress spanning days to weeks. Furthermore, the correlation strength between the functional state index and the psycho-emotional index is not static; it fluctuates based on the nature of the stressor. This dynamic interplay offers a nuanced understanding of the physiological responses under varying conditions.

While this study has laid the groundwork for understanding the physiological cost of activities in military personnel, there remains significant scope to delve deeper into the long-term implications of continuous monitoring. It is imperative to discern how such persistent data collection and analysis might influence the mental well-being of soldiers and even more so—their quality of life. This can pave the way for a more holistic approach to military readiness, where physical and mental health are given equal precedence. Further studies can also explore the potential of integrating other physiological markers with the ECG data to provide a more comprehensive picture of a soldier's readiness and overall health. The proposed technology, while promising, can be further refined and adapted for different military scenarios and environments. Exploring its applicability in diverse settings can be a focal point of subsequent research endeavors.

The proposed technology is a promise in military readiness assessment. It offers an objective measure of combat readiness and provides insights into the physiological and emotional well-being of military personnel. As we move forward, it is crucial to expand upon this foundation, ensuring that our military personnel are physically ready and mentally resilient to face the challenges that lie ahead.

## Data Availability

The raw data supporting the conclusions of this article will be made available by the authors, without undue reservation.
